# Synergistic Protection of Combined Probiotic Conditioned Media against Neonatal Necrotizing Enterocolitis-Like Intestinal Injury

**DOI:** 10.1371/journal.pone.0065108

**Published:** 2013-05-24

**Authors:** Sheng-Ru Shiou, Yueyue Yu, Yuee Guo, Shu-Mei He, C. Haikaeli Mziray-Andrew, Jeanette Hoenig, Jun Sun, Elaine O. Petrof, Erika C. Claud

**Affiliations:** 1 Department of Pediatrics, Section of Neonatology, The University of Chicago, Chicago, Illinois, United States of America; 2 Department of Medicine, Section of Infectious Diseases and GIDRU, Queen's University, Kingston, Ontario, Canada; 3 Department of Pediatrics, Section of Pediatric Gastroenterology and Nutrition, Southern Illinois University School of Medicine, Carbondale, Illinois, United States of America; 4 Edward Hospital, Naperville, Illinois, United States of America; 5 Department of Biochemistry, Rush University, Chicago, Illinois, United States of America; 6 Department of Medicine, Section of Gastroenterology, The University of Chicago, Chicago, Illinois, United States of America; Emory University School of Medicine, United States of America

## Abstract

Balance among the complex interactions of the gut microbial community is important for intestinal health. Probiotic bacteria can improve bacterial balance and have been used to treat gastrointestinal diseases. Neonatal necrotizing enterocolitis (NEC) is a life-threatening inflammatory bowel disorder primarily affecting premature infants. NEC is associated with extensive inflammatory NF-κB signaling activation as well as intestinal barrier disruption. Clinical studies have shown that probiotic administration may protect against NEC, however there are safety concerns associated with the ingestion of large bacterial loads in preterm infants. Bacteria-free conditioned media (CM) from certain probiotic organisms have been shown to retain bioactivity including anti-inflammatory and cytoprotective properties without the risks of live organisms. We hypothesized that the CM from *Lactobacillus acidophilus (La), Bifidobacterium infantis (Bi)*, and *Lactobacillus plantarum (Lp)*, used separately or together would protect against NEC. A rodent model with intestinal injury similar to NEC was used to study the effect of CM from Lp, La/Bi, and La/Bi/Lp on the pathophysiology of NEC. All the CM suppressed NF-κB activation via preserved IκBα expression and this protected IκBα was associated with decreased liver activity of the proteasome, which is the degrading machinery for IκBα. These CM effects also caused decreases in intestinal production of the pro-inflammatory cytokine TNF-α, a downstream target of the NF-κB pathway. Combined La/Bi and La/Bi/Lp CM in addition protected intestinal barrier function by maintaining tight junction protein ZO-1 levels and localization at the tight junction. Double combined La/Bi CM significantly reduced intestinal injury incidence from 43% to 28% and triple combined La/Bi/Lp CM further reduced intestinal injury incidence to 20%. Thus, this study demonstrates different protective mechanisms and synergistic bioactivity of the CM from different organisms in ameliorating NEC-like intestinal injury in an animal model.

## Introduction

Necrotizing enterocolitis (NEC) is a life-threatening inflammatory bowel disorder that affects approximately 10% of premature infants born <1500 gm. NEC carries a mortality rate of 20–30% and there is no specific treatment [1]–[4]. The major risk factors are prematurity, enteral feeding, and bacterial colonization [5]–[7]. It has been hypothesized that the immature intestinal barrier of the preterm gut results in increased translocation of intestinal microbiota across the epithelium. In response to bacterial stimulation, increased NF-κB signaling of the immature enterocyte leads to exaggerated production of inflammatory cytokines, resulting in further barrier disruption and intestinal necrosis which causes the signs and symptoms of disease [7]–[10].

Probiotics are bacteria that have beneficial health effects beyond their inherent nutritional value. Probiotics have been used to treat a variety of gastrointestinal diseases, including inflammatory bowel diseases (IBD), irritable bowel syndrome, and pouchitis [11]–[13]. The mechanisms by which probiotics protect against intestinal diseases include optimization of microbial balance, competitive exclusion of pathogens, promotion of mucus secretion and bacteriocin production, enhancement of barrier integrity, and maturation of intestinal immunity [14].

Select probiotics have specifically been shown to protect intestinal integrity and reduce experimental NEC incidence in animal models. In particular *Bifidobacteria* and *Lactobacilli* are gram-positive anaerobic bacteria that colonize and are the predominant organisms in the gastrointestinal track of healthy breast-fed infants [15]. These bacteria are less prevalent in formula-fed and premature infants, who have the highest risk of NEC [16], [17]. It has been shown that oral administration of *Bifidobacterium bifidum, Lactobacillus bulgaricus, Lactobacillus reuteri, Lactobacillus rhamnosus* GG (LGG) can protect against mechanisms associated with the pathophysiology of NEC in the developing gut or in animal models of NEC [18]–[24]. The underlying mechanisms involve reduced apoptosis, improved intestinal barrier integrity, and suppressed inflammation. These results support the concept that select probiotics may elicit distinct protective effects and are potential valuable treatments to prevent NEC. In humans, combined administration of the specific probiotics *Lactobacillus acidophilus* (La), *Bifidobacterium infantis* (Bi), and *Bifidobacterium bifidum* live bacteria has been shown to significantly reduce NEC incidence in infants in prospective clinical studies [25]–[27]. These studies, while documenting possible beneficial clinical effects did not investigate possible mechanisms for the protective effect of the probiotics used.

Several reports in the literature have raised safety concerns over the practice of ingesting large bacterial loads, especially in sick and immunocompromised patients [28]–[31]. While some protective effects of probiotics require direct live bacterial-epithelial cell interaction, not all do. The probiotic conditioned medium (CM) is the broth which has been used to grow the probiotic bacteria subsequently filtered to remove all bacteria. In contrast to live probiotics, CM does not carry risk of infection and sepsis and may be relatively safer for clinical use. Others and we have previously demonstrated that certain probiotics secrete bioactive factors into CM which have both anti-inflammatory and cytoprotective properties [32], [33]. Studies have suggested that secreted products from the specific bacteria used in the human probiotic clinical trials in NEC may address some of the inflammatory and intestinal barrier disruption insults associated with NEC. CM from heat-killed La with its culture supernatant has been shown to protect tight junctions of HT-29 cells from aspirin-induced damage [34]. CM from Bi has been shown to preserve epithelial cell barrier function in the context of IFN-γ and TNF-α induced injury by enhancing the tight junction proteins ZO-1 and occludin expression in T84 colonocytes. In a spontaneous colitis model using IL-10^-/-^ mice, oral administration of Bi CM attenuated inflammation and protected colonic barrier function [35]. Additionally, we have previously demonstrated that Lp CM inhibits NF-κB activation as well as release of proinflammatory cytokines and chemokines in response to multiple proinflammatory stimuli [32]. We thus hypothesized that the CM from La, Bi, and Lp, used separately or together would protect against the pathophysiology of NEC. In order to test our hypothesis, we have adopted a previously described animal model that manifests intestinal injury similar to human NEC [36]. We recognize that the natural history and thus pathophysiology of human NEC in preterm babies may not be exactly the same as what is incurred by this model and therefore the term “NEC” used in association with our animal study refers to NEC-like intestinal injury. Our data demonstrate that double combined La/Bi CM significantly reduced intestinal injury incidence and triple combined La/Bi/Lp CM further reduced intestinal injury incidence, suggesting a synergistic effect of CM from separate bacterial organisms in ameliorating intestinal injury in an animal model. Lp CM as well as La/Bi CM suppressed NF-κB activation via preserved IκBα expression and this protected IκBα expression was associated with decreased liver activity of the proteasome that plays an important role in regulated IκBα protein levels. La/Bi and La/Bi/Lp CM also protected IEC barrier function by maintaining ZO-1 at the cellular tight junctions. CM from select probiotic bacteria chosen for specific anti-inflammatory and cytoprotective effects relevant to the pathogenesis of NEC may thus constitute a potentially novel treatment for preventing this disease in vulnerable preterm infants.

## Materials and Methods

### Ethics statement

This study was carried out in strict accordance with the recommendations in the Guide for the Care and Use of Laboratory Animals of the National Institutes of Health. All animal work was conducted under the animal protocol No. 71557 and was approved by the University of Chicago Institutional Animal Care and Use Committee (IACUC). Cesarean-section was performed under isoflurane anesthesia and all efforts were made to minimize suffering. If a rat pup showed illness during the course of the study, the animal was humanely euthanized and death was not used as an endpoint.

### Preparation of bacterial conditioned media

The *Lactobacillus plantarum*, *Lactobacillus acidophilus*, and *Bifidobacteria infantis* used were from ATCC (No. 14917, 53544 and 15697, respectively). Bacteria were first grown and expanded in MRS broth (DeMan Rogosa & Sharpe, Difco) at 37°C and 5% CO2 under anaerobic and non-agitating conditions, centrifuged (20 min, 4,500×g), and resuspended in MRS (*L. plantarum*) or modified Hank's balanced saline solution (HBSS) (*L. acidophilus* and *B. infantis*) supplemented with 0.04 M MgSO4, 0.03 M MnSO4, 1.15 M K2PO4, 0.36 M sodium acetate, 0.88 M ammonium citrate, 10% polysorbate (growth factor for *Lactobacillus* sp) and 20% dextrose. Bacteria then were propagated overnight at 37°C, 5% CO2 under nonagitating conditions to 2×109 cfu/mL. The culture was again centrifuged and the supernatant was aseptically filtered using 0.22 µm low protein binding cellulose acetate filters (Millipore) and used as conditioned media.

### Neonatal rat experimental intestinal injury model

All animal studies were reviewed and approved by the Institutional Animal Care and Use Committee. Animal experiments were conducted following a well described rat pup experimental intestinal injury animal model [36]. In this model, pups were subjected to major risk factors for human NEC (prematurity, formula feeding, bacterial colonization, and hypoxia-ischemia), resulting in changes in the immature rat pups similar to those found in humans as follows: the abdomen was distended, blood was detected in the stool, and the ileum and proximal colon were the most affected parts of the intestine [37].

Neonatal rats from time-dated pregnant Sprague-Dawley dams were delivered by cesarean section at E20 following isoflurane anesthesia. Pups were then stabilized, dried, and maintained in a humidified incubator at 37°C and bowel/bladder function was stimulated by a soft cotton-tip applicator. Pups were fed with Esbilac puppy formula every 3 hours via an orogastric feeding catheter and colonized with 10^7^ CFU of *Serratia marcescens*, *Klebsiella pneumoniae*, and *Streptococci viridans* in 100 µl formula once daily. Pups were also stressed under 5% O_2_+95% N_2_ for 10 min after feeding three times a day. The feeding volume began at 0.1 ml and was increased incrementally up to 0.25 ml. Naturally born rat pups fed by dams were included as healthy controls. For conditioned medium (CM) groups, CM from Lp, La and Bi were used in different combinations (Lp, La/Bi, or La/Bi/Lp CM) and compared to the experimental control category of vehicle alone. Fifty µl CM was administered once daily 3 hours before administration of *Serratia marcescens*, *Klebsiella pneumoniae*, *Streptococci viridans*. Formula (regular volume minus 50 µl) was fed immediately after CM administration.

Animals were sacrificed when ill or at the end of the experiment on day 5. The health of animals was evaluated by morbidity scale: score 0: active, pink color, no abdominal distension or discoloration, score 1: lethargic, pink color, no abdominal distension or discoloration, score 2: not active, facial cyanosis, slight abdominal distension, score 3: not active, facial and peripheral cyanosis, abdominal distension with or without abdominal discoloration, normal stools, score 4: not active, marked cyanosis, abdominal distension, abdominal discoloration, bloody stools, score 5: death. Animals that reached score 3 were considered ill and sacrificed. The intestines were collected and fixed in 10% buffered formalin overnight for tissue section preparation. H&E-stained intestinal sections were assessed histologically for ileal damage by a pathologist blinded to treatment groups using a published scoring system to evaluate the degree of intestinal injury on a “0–4” scale as follows: 0 no histological damage; 1 (mild), slight submucosal and/or lamina propria separation; 2 (moderate), moderate separation of the submucosa and/or lamina propria and/or edema in the submucosa and muscular layers; 3 (severe), severe separation of the submucosa and/or lamina propria and/or severe edema in the submucosa and muscular layers with regional villous sloughing; and 4 (necrosis), loss of villi and necrosis. Scores ≥2 were defined as NEC-like intestinal injury [36].

The NEC-like intestinal injury incidence of the animal model performed in our animal facility has been consistent and similar to that of other groups using this model [38], [39]. Based on power and sample size calculation using Stat software to compare two proportions (percentages), 45 animals in each group are needed to document a 30% decrease in the incidence of the disease with an α of 0.05 and a power of 80%. Similar or higher numbers of animals were included in each group to test the efficacy of CM treatment.

### *Ex vivo* intestinal barrier function assay

To assess barrier integrity of different intestinal regions, a previously described *ex vivo* intestinal loop assay was performed [40], [41]. Explants of ileum (right above and next to cecum) and jejunum/duodenum (at least 5 cm away from ileum) were harvested from experimental rat pups. Explanted intestinal segments were flushed with dialyzed 10 kDa FITC-dextran (1 mg/mL in PBS, Sigma Aldrich), ligated with sutures at one end, filled with the same FITC-dextran solution, and ligated at the other end. Explanted loops were rinsed twice in PBS, transferred into wells containing 0.6 mL PBS, and left at RT. One hundred and forty µl PBS from the wells were sampled at 30, 60, and 90 minutes after explants were placed in PBS and added to 96-well plates in duplicate (60 µl/well) to determine FITC-dextran flux across the loops. Fluorescence intensity (excitation at 490 nm and emission at 525 nm) was measured using a Synergy-2 fluorometer (Bio-Tek Instruments, Winooski, VT). FITC-dextran flux/concentration (µg/ml) was calculated with standard curves and then normalized to intestinal length (cm). Barrier integrity of paired ileum and jejunum/duodenum from same animals (n = 5) at all time points was measured and compared to ileum.

### *In vivo* intestinal barrier function assay

To investigate intestinal lumen-to-blood permeability/barrier function in the NEC-like intestinal injury model, an *in vivo* permeability assay was performed using FITC-dextran as described previously [42]. Previous publications have used both 4- and 10-kDa FITC-dextran to determine *in vivo* gut permeability [43]–[46]. Immature intestine is considered leakier than mature intestine [47] and a small molecule tracer does not allow for detection of subtle alterations in barrier function. After optimization, a 10-kDa rather than a 4-kDa FITC-dextran (Sigma Aldrich) was used in our study to better distinguish improved barrier function of leaky immature intestine. At the end of the 5-day intestinal injury model, surviving pups grossly have histologically normal intestine (score “0”). Thus, these pups were used to evaluate barrier function in order to avoid the confounding effect of barrier disruption due to intestinal necrosis associated with disease state.

Briefly, all surviving rat pups at the end of the experiments on day 5 were starved for 4 hours and then received 10 mg/ml 10-kDa FITC-dextran (40 mg/100 g body weight) via an oral gavage tube directly into the stomach. Four hours later, whole blood was collected and centrifuged at 1,500×g for 10 minutes. Twenty five µl of serum was diluted in 100 µl of PBS and the diluted serum was added in a 96-well black wall microplate in duplicate (60 µl per well) for measurement of fluorescence intensity (excitation at 490 nm and emission at 525 nm) using a Synergy-2 fluorometer (Bio-Tek Instruments, Winooski, VT). Standards were included to determine the concentrations of FITC-dextran in the serum. High concentrations of FITC-dextran in the serum indicate greater transmucosal transport of FITC-dextran across the intestinal barrier to blood or poorer barrier function.

### Immunohistochemistry staining

Intestinal (ileal) segments were formalin fixed and paraffin embedded. Sections were deparaffinized in xylene and hydrated with ethanol. For antigen unmasking, slides were heated in 10 mM sodium citrate buffer (pH 6.0) before treatment with 0.3% hydrogen peroxide. The specimens were blocked with 5% BSA in TBST (Tris-buffered saline with 0.05% vol/vol Tween-20) followed by incubation at 4°C overnight with p-NF-κB, IκBα antibodies (Cell Signaling), or control IgGs isolated before immunization from the same animal species used to generate the p-NF-κB or IκBα antibodies. After washing, the sections were incubated with polymer-HRP secondary antibodies (Dako, Carpinteria, CA). Positive staining and nuclei were visualized with DAB chromogen and hematoxylin counterstaining, respectively. For quantitation of NF-κB activity, nuclear p-NF-κB positive versus total IEC from three fields of each slide (n = 3) were counted and presented as mean of percentages of p-NF-κB-positive over total IEC ± SE. For quantitation of IκBα staining, H&E intensity was scored on a “0” to “4” scale with “0” being the lowest and “4” being the highest intensity among all samples examined [48]. Results are presented as mean of scores ± SE.

### Immunoblot Analysis

Immunoblotting was conducted as described previously [49]. For tissue lysate preparation, 2-cm distal intestine (ileum) was homogenized with a hand-held homogenizer (Pellet Pestle, Kimble/Kontes, Vineland, NJ) in lysis buffer (50 mM Tris⋅HCl pH 7.4, 150 mM NaCl, 1 mM EDTA, 1% SDS, 50 mM DTT, 50 µg/ml aprotinin, 50 µg/ml leupeptin, and 5 mM PMSF). Lysates were sonicated and then centrifuged at 10,000×g for 10 minutes at 4°C. The protein concentration of supernatants was determined using the BCA protein assay kit (Pierce). Lysates were resolved by SDS-PAGE and transferred to PVDF membranes. Membranes were blocked in 5% milk PBS-T [0.1% Tween 20 (v/v) in PBS] for 1 h at RT and then probed with primary and appropriate secondary antibodies in 5% milk PBS-T. Immunoreactive bands were visualized by chemiluminescence reaction using ECL reagents (Amersham Biosciences) followed by exposure of the membranes to autoradiography film (Midsci, St. Louis, MO). Protein levels were quantified by densitometry using ImageJ software. The IκBα and GAPDH antibodies were purchased from Transduction Laboratories, Inc. (San Diego). The occludin antibody was obtained from Invitrogen (Grand Island, NY).

### Expression and purification of mUIM-2 for proteasome purification

pET15b-mUIM-2 plasmid, kindly provided by Dr. Lenore K. Beitel [50], was transformed into *Escherichia* coli BL21 (DE3) competent cells by heat shock. The bacteria were then grown in Luria-Bertani (LB) broth containing 100 ug/ml ampicillin to an optical density (O.D.) of 0.6 (A600 nm). The culture was then induced with 0.25 mM IPTG for 16 hours and harvested by centrifugation at 3000×g for 15 minutes at 4°C. Culture pellets were resuspended in buffer A (50 mM HEPES, pH 7.5, 300 mM NaCl, 25 mM imidazole (Fisher Scientific, Ottawa, ON), lysed by sonication, and centrifuged at 30,000×g for 30 minutes at 4°C. The resulting supernatant was loaded to a Ni-NTA column, and the His(6)-tagged mUIM-2 was eluted with a gradient from 25 mM to 500 mM imidazole. The fractions eluted were then analyzed with 12% SDS-PAGE gels followed by Coomassie Blue G-250 (Fisher Scientific) staining. Fractions containing pure mUIM-2 were pooled and subsequently concentrated with Millipore Amicon Ultra centrifugal devices (10,000 MWCO).

### Expression and Purification of GST-UBL for proteasome purification

The pGEX-2T-UBL plasmid, kindly provided by Juli Feigon [51], was expanded and expressed as in the mUIM-2 purification except that the bacteria were induced with IPTG for 4 hours instead of 16 hours. After 4 hours at 37°C, the bacteria were harvested by centrifugation at 3000×g at 4°C for 15 minutes. The cell pellets were then resuspended in purification buffer (50 M HEPES, pH 7.5, 5 mM MgCl_2_, 10% glycerol (Fisher Scientific)) and lysed by sonication. The lysed cells were then centrifuged at 30,000×g at 4°C for 30 minutes and the supernatant was loaded onto Glutathione-Agarose beads, with gentle agitation at 4°C for 2 hours. The agarose beads were centrifuged at 500×g for 2 minutes, supernatant was removed, and beads were washed with 10 column volumes of purification buffer to obtain GST-UBL.

### Proteasome purification from rat livers

Proteasomes from experimental rat livers were purified according to T.C. Scanlon et al and H.C. Besche et al with some modifications [50], [52]. The ubiquitin-like (UBL) domain of the proteasome associated protein hHR23B has high affinity for the 26S proteasome and GST-UBL served as the affinity matrix. After binding to the UBL domain, proteasomes were eluted with an excess of a recombinant His-tagged ubiquitin-interacting motif (UIM). The UIM has a higher affinity for UBL than the proteasomes and competes with the 26S proteasome for UBL binding, allowing proteasomes to be eluted from the GST-UBL beads. The His tag then allows subsequent removal of any excess UIM from the purified proteasome preparation through use of Ni-NTA beads as described below.

In brief, 300 mg to 1 g of rat livers were homogenized in purification buffer (50 mM HEPES, pH 7.5, 5 mM MgCl2, 10% glycerol) supplemented with 2 mM ATP and protease inhibitors (Roche, Laval, QC) and centrifuged at 21,100×g for 1 hour at 4°C. The supernatants were then added to the affinity matrix (GST-UBL beads), and the suspension was incubated at 4°C for 4 hours with rotation. The beads were then washed with 10 column volumes of purification buffer containing 2 mM ATP. Proteasomes bound to the GST-UBL matrix were eluted by addition of 20-fold molar excess of purified mUIM-2 and subsequent overnight incubation with continuous rotation at 4°C. Five hundred microlitres of Ni-NTA agarose beads were added to the suspension and incubated at 4°C for 2 hours to remove excess mUIM-2. The supernatants containing the purified proteasomes were then collected by centrifuging at 500×g for 3 minutes and subjected to a BCA assay (Pierce, Nepean, ON) to determine protein concentration.

### 26S Proteasome activity assay with purified proteasome

Chymotrypsin-like activity of the 26S proteasome was measured using a Varian Cary Eclipse fluorescence spectrophotometer by a fluorogenic substrate assay. In brief, 25 µg of affinity-purified proteasome was incubated in assay buffer (25 mM HEPES/pH 7.6, 10 mM EDTA, 0.03% SDS, 2 mM ATP) supplemented with 65 µM of suc-leu-leu-val-tyr-AMC (SLLVY-AMC) as the substrate. Proteasome chymotrypsin-like activity was determined by measuring the fluorogenic signal generated by cleavage and release of the fluorogenic compound AMC (7-amino-4-methylcoumarin) over time. Fluorescence generated from free AMC was monitored every minute for 30 minutes over the linear range of the reaction (Excitation: 380 nm, emission: 460 nm), and proteasome activity was determined by calculating the rate (relative fluorescence units/minute or RFU/minute) of free AMC released over time using Varian Cary Eclipse kinetics application software. Experiments were performed with 96-well half area black polystyrene plates (Corning Incorporated, Corning, NY) in quadruplicates. Epoxomicin (Enzo Life Sciences Inc, Farmingdale, NY), a naturally occurring selective proteasome inhibitor [53] was added to separate wells to 5 µM. Epoxomicin inhibition of proteasome activity served as a control to confirm specificity of the assay.

### Analysis of TNF-α in the intestine

Local production of TNF-α, an inflammatory cytokine elevated in human and animal NEC, was analyzed from whole intestinal tissues. Intestinal tissues in RIPA buffer were homogenized by sonication and cleared by centrifugation. Protein concentration of the resulting supernatant/intestinal lysates was determined by BCA protein assay (Pierce, Nepean, ON). Fifty µg of lysate was used to determine intestinal TNF-α levels using the xMAP technology, a flow-assisted and bead-based Cytokine Assay (Bio-Rad) and analyzed with five-parameter curve fitting generated standard curve using the Bioplex Manager 6.0 software (Bio-Rad). The cytokine concentration in lysates is expressed as picograms cytokine per mg of intestinal lysate (pg/mg).

### Immunofluorescence Staining of ZO-1

After deparaffinization and rehydration, intestinal sections were blocked in 5% BSA for 1 h at RT and then incubated with ZO-1 and occludin antibodies (Invitrogen, Grand Island, NY.) at 1200 dilution, followed with an Alexa Fluor 488-conjugated second antibody (Molecular Probes, Eugene, OR). Nuclei were labeled with DAPI (100 ng/ml). Images were acquired by confocal microscopy using Leica SP2.

### Analysis of mRNA expression by quantitative real-time RT-PCR

RNA was isolated from frozen intestine using RNeasy Plus Mini Kit (Qiagen, Valencia, CA) according to the manufacturer's protocols. One µg total RNA was reverse transcribed in 20 µl using RNA to cDNA EcoDry Premix (Double primed) (Clontech, Mountain View, CA). TaqMan real-time PCR (qPCR) was performed in triplicate using 0.2 µl cDNA and primers and probes from Integrated DNA Technologies, Inc (Coralville, Iowa) on a 7900 Detector (Applied Biosystems, Foster City, CA). The primers and probe used to detect expression of rat ZO-1 (NM_001106266) are 5′-GAAAGGTAAGGGACTGGAGATG-3′, 5′-CATATCCTCCTTACTCACCACAA-3′, and 5′-/56-FAM/AGACAGCCT/ZEN/CTCAACAGAAAGCAGAAG/3IABkFQ/-3′. The primers and probe used to detect expression of rat GAPDH (NM_017008) are 5′-GTAACCAGGCGTCCGATAC-3′, 5′-GTTCTAGAGACAGCCGCATC-3′, and 5′-/56-FAM/CGTTCACAC/ZEN/CGACCTTCACCATCTT/3IABkFQ/-3′. The threshold cycle (Ct) value for each well was obtained using the instrument's default setting. Gene expression was normalized and calculated using the ΔΔCt method: 2^-ΔΔCt^ = 2^–[Ct (ZO-1) –Ct (GAPDH)]^ experiment/2^–[Ct (ZO-1) –Ct (GAPDH)]^ dam-fed and presented as fold changes to the dam-fed healthy control.

### Statistical analysis

All data are presented as means ± SE. Statistical analysis was performed using the χ2 test for NEC-like intestinal injury incidence, Student's *t* test for paired data, or one-way analysis of variance (ANOVA) with a Bonferroni correction for multiple comparisons with GraphPad InState software. Differences were considered to be significant with p values <0.05.

## Results

### Combined conditioned media (CM) from *Lactobacillus plantarum* (Lp), *Lactobacillus acidophilus* (La), and *Bifidobacterium infantis* (Bi) synergistically reduced intestinal injury incidence

**Figure 1:**
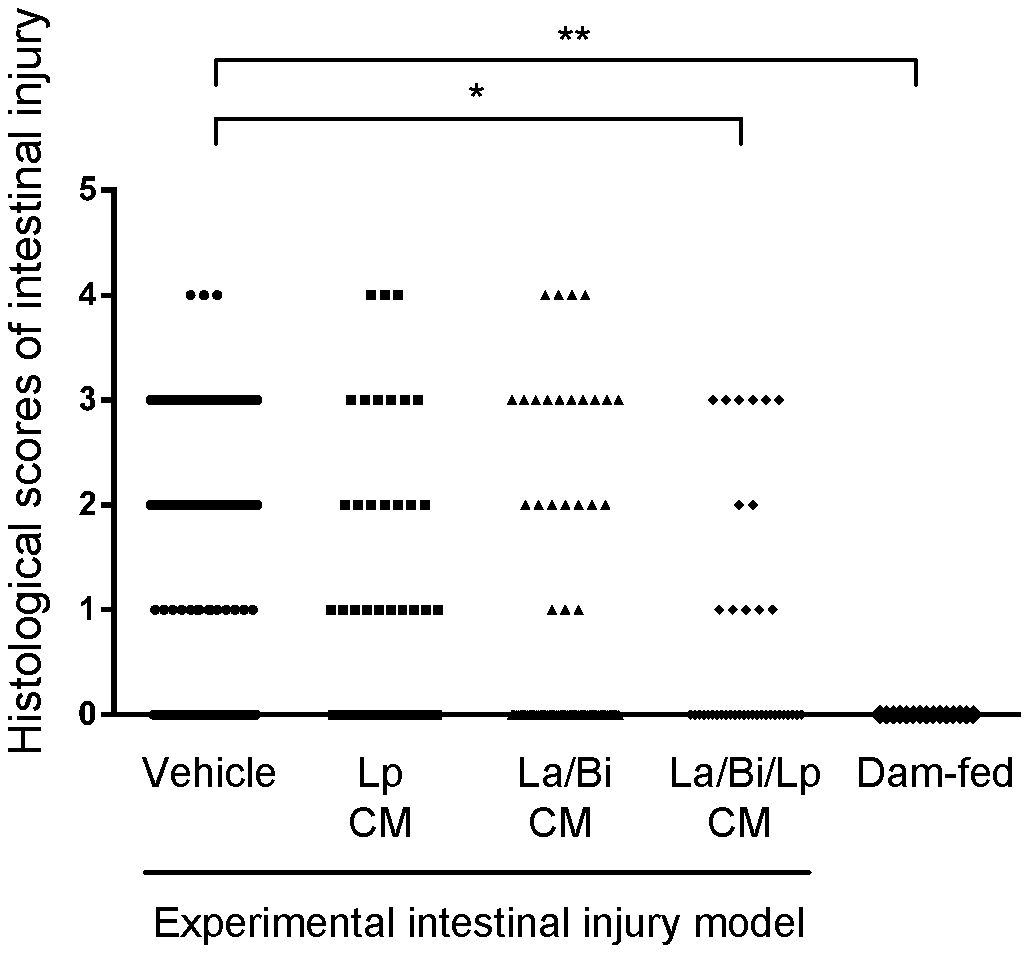
Histological scores of intestinal injury. An animal model with intestinal injury similar to NEC was performed as described in “Materials and Methods”. Intestine from healthy dam-fed controls and experimental pups treated with vehicle or different CM were H&E stained and intestinal injury was evaluated on a “0–4” scale by a pathologist blinded to experiments. The healthy dam-fed pups show no intestinal injury and the three combined CM significantly decreased severity of the intestinal injury. “*” and “**” depict p<0.05 and 0.001, respectively, by one-way ANOVA with Bonferroni correction.

An experimental rodent model with intestinal injury similar to NEC was used to examine the effect of oral administration of CM from Lp, La and Bi. Non-stressed and naturally born dam-fed pups served as healthy controls. Vehicle treatment alone served as an experimental control. Pups undergoing animal studies were fed with formula every 3 hours via an orogastric feeding tube. Due to this nature of the animal model, only certain numbers/categories of animals were included in one experiment. The vehicle-treated control group was always included in experiments, thus has a greater number of animals compared to other groups. The degree of intestinal injury was scored on a “0–4” scale and score ≥2 is defined as diseased injury. All dam-fed healthy controls had score “0”. In the triple combined La/Bi/Lp CM, the histological scoring of intestinal damage shows no score 4 (the most severe injury) and statistical analysis confirmed that this combined CM significantly reduced disease severity when compared with vehicle-treated group (Fig. 1). Oral administration of Lp CM and La/Bi CM reduced intestinal injury incidence (score ≥2) from 43% to 32% (n = 50, p = 0.1780) and to 28% (n = 70, p = 0.0433), respectively (Table 1). Combined CM from all three organisms further reduced NEC-like intestinal injury incidence from 43% to 20% (*n* = 41, p = 0.0064).

**Table 1:** Combined conditioned media of probiotics lowered intestinal injury incidence.

	Diseased	Non-diseased	Total
Experimental model + vehicle	65 (43%)	87	152
Experimental model + Lp CM	16 (32%)	34	50
Experimental model + La/Bi CM	20 (28%)*	50	70
Experimental model + La/Bi/Lp CM	8 (20%)*	33	41
Dam-fed	0 (0%)	30	30

A neonatal rat intestinal injury model and disease scoring were conducted as in the “Materials and Methods”. Experimental model rat pups were treated with vehicle or different combination of conditioned media (CM) from *Lactobacillus plantarum* (Lp), *Lactobacillus acidophilus* (La), and *Bifidobacterium infantis* (Bi). Naturally born and dam-fed pups were healthy controls. “*” depicts p<0.05 compared to the vehicle-treated group between the experimental model groups by χ2 test.

### Conditioned media from Lp, La, and Bi decreased NF-κB activation and maintained IκBα expression

**Figure 2:**
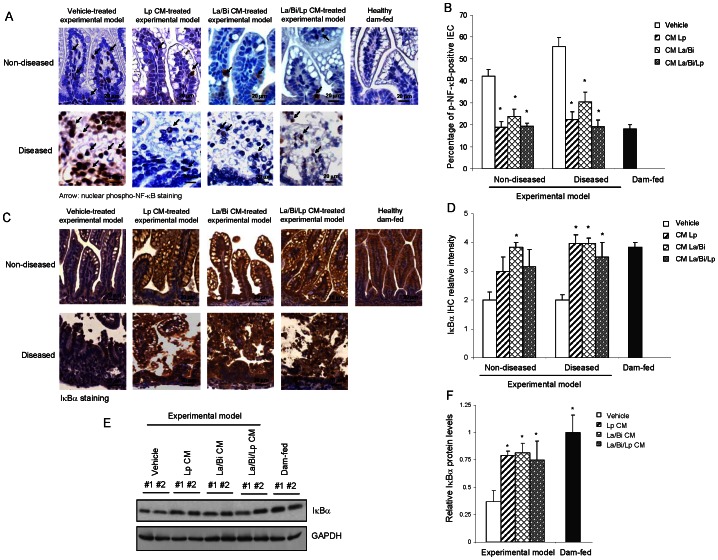
CM from *L. plantarum*, *L. acidophilus*, and *B. infantis* suppressed NF-κB signaling and preserved IκBα expression. Intestines from healthy dam-fed controls and experimental intestinal injury pups treated with vehicle or different CM were IHC stained for the active/phosphorylated form of NF-κB p65 (p-NF-κB) and IκBα or lysed for immunoblotting for IκBα. ***A.*** Representative IHC staining (at 400×) shows that CM decreased IEC NF-κB activity/nuclear p-NF-κB (arrows) in experimental pups with or without intestinal injury (n = 3 per group). ***B.*** NF-κB activity from ***A.*** was quantified by percentage of nuclear p-NF-κB-positive IEC in three fields of each intestinal section (n = 3). ***C.*** Representative IHC staining shows La/Bi CM and all CM protection of IEC IκBα expression in experimental pups with or without intestinal injury, respectively, (n = 3 per group). ***D.*** IHC intensity of IκBα from ***C.*** was scored on a 0–4 scale to relatively quantify levels of IκBα. ***E.*** Immunoblotting shows CM protection of IEC IκBα expression in experimental pups. Intestines of vehicle- and CM-treated experimental pups with disease and healthy dam-fed pups were lysed and subjected to IκBα immunoblotting. IκBα protein levels were quantified by densitometry using ImageJ software and normalized to GAPDH and presented in ***F.*** Results are presented as mean of percentages, scores, or relative density ± SE. “*” depicts p<0.05 compared to the vehicle group by one-way ANOVA with Bonferroni correction.

As the intestinal inflammation in NEC has been shown to correlate with activation of NF-κB [54], IHC staining of the active/phosphorylated form of NF-κB p65 (p-NF-κB) was used to determine if CM protection was associated with altered NF-κB signaling. As in Figure 2A and B, increased numbers of p-NF-κB-positive intestinal epithelial cells (IEC) are readily observed in the intestines of vehicle-treated non-diseased experimental pups (42.2%) compared to healthy dam-fed control pups (18.2%). Elevated p-NF-κB-positive cell numbers are further increased in vehicle-treated diseased (score ≥2) experimental model pups (55.6%). These results are consistent with previous findings demonstrating importance of NF-κB activation during the development of the disease [54]. In both diseased and non-diseased groups, Lp, La/Bi, and La/Bi/Lp CM significantly decreased p-NF-κB-positive cell numbers (Fig. 2A and B). Whether CM suppression of NF-κB activity was associated with enhanced expression of the NF-κB inhibitor IκBα was determined by IHC. IκBα was readily detected in the intestines of healthy dam-fed pups, but was diminished in vehicle-treated experimental model intestines with or without disease (Fig. 2C). La/Bi CM protected IκBα in non-diseased experimental model pups and all the CM protected IκBα expression in diseased experimental model pups, suggesting that the anti-inflammatory property of Lp, La, and Bi CM can be mediated through protected IκBα expression (Fig. 2C and D). IκBα levels in the intestine evaluated by immunoblotting and densitometry confirm H&E results that CM protected IκBα expression in the intestinal injury model (Fig. 2E and F).

### Conditioned media from Lp, La, and Bi decreased proteasome activity

**Figure 3:**
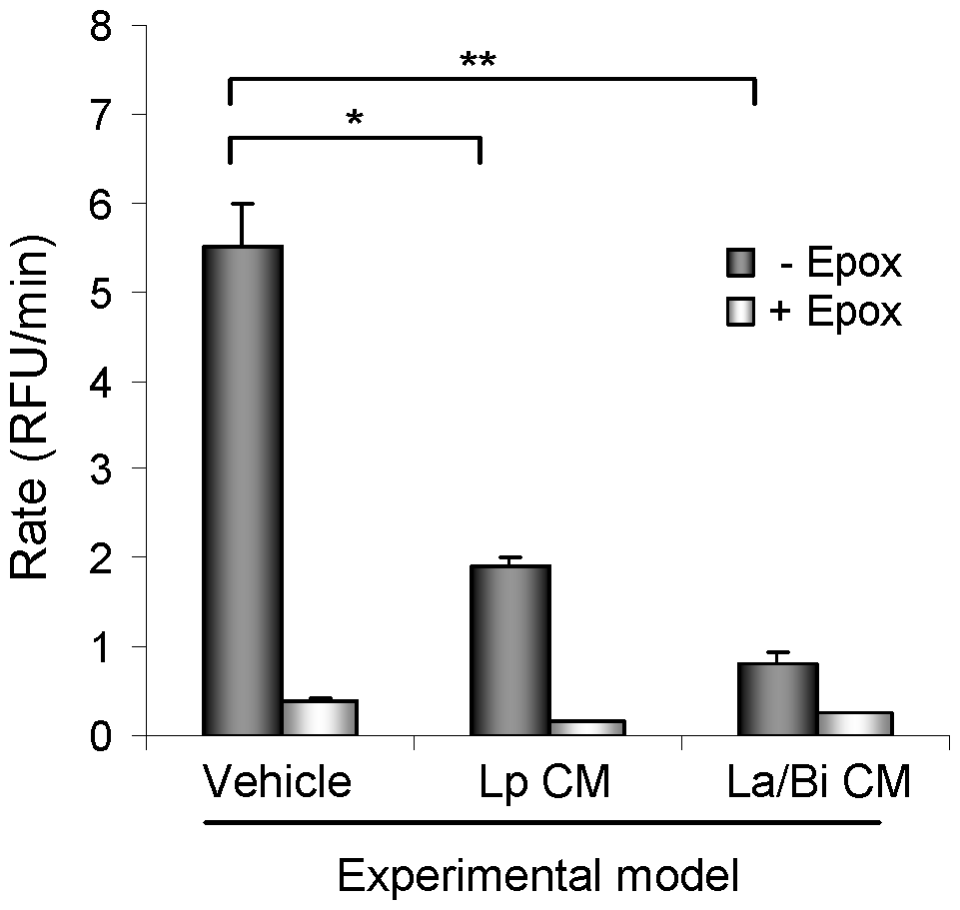
CM from *L. plantarum* (Lp CM) and *L. acidophilus* and *B. infantis* (La/Bi CM) inhibited proteasome activity. CM inhibited proteasome activity in the experimental intestinal injury model. At the end of each experiment, liver was harvested, lysed, and proteasome was purified and subjected to a proteasome chymotrypsin-like activity assay as described in “Materials and Methods”. Five µM of the proteasome specific inhibitor epoxomicin (Epox) was added in separate wells as a control to confirm specificity of the assay. Proteasome chymotrypsin-like activity was determined by the rate of generation of fluorogenic product over time. Five µM of Epox was added in separate wells as a control to confirm specificity of the assay. Data (n = 4) are presented as mean ± SE. “*” and “**” depict p<0.05 and 0.001 compared to vehicle groups, respectively, by one-way ANOVA with Bonferroni correction.

IκBα is degraded by the chymotrypsin-like (CTL) activity of the proteasome, allowing release and activation of NF-κB [55]; hence, the effect of CM on chymotrypsin-like proteasome activity was investigated. Colitis has been demonstrated to globally affect proteasome activity in non-gastrointestinal tissues [56]. Among organs tested for proteasome activity in neonatal rats, liver tissues provided the optimal yield and activity (data not shown). Therefore, the effect of CM on proteasome activity was assessed using liver homogenates. In liver homogenates from the experimental model, the vehicle-treated group had the highest proteasome activity. Gavage with Lp and La/Bi CM significantly reduced liver proteasome activity in the experimental model (Fig. 3). Epoxomicin, a naturally occurring proteasome inhibitor was used in parallel and its inhibition of proteasome served as a positive control to confirm specificity of the assay.

### Conditioned media from Lp, La, and Bi decreased local production of TNF-α

**Figure 4:**
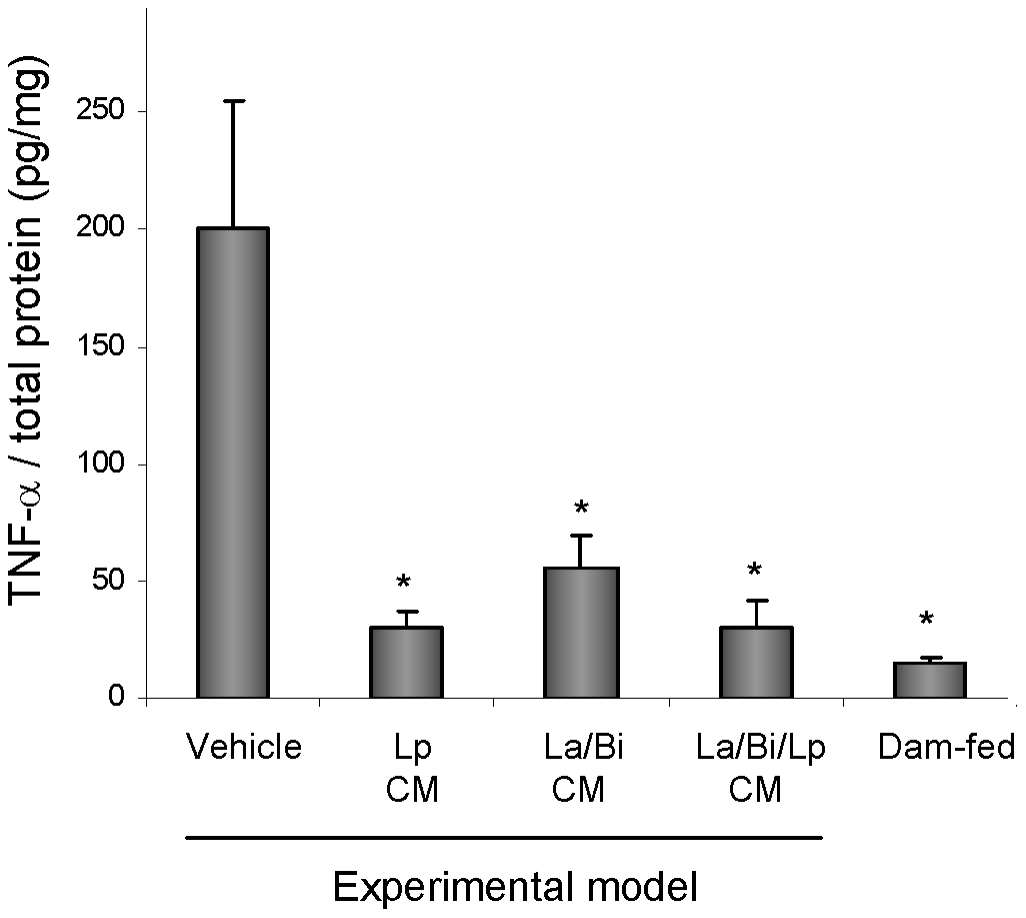
CM from *L. plantarum*, *L. acidophilus*, and *B. infantis* decreased the pro-inflammatory cytokine TNF-α in the intestine. Intestine was collected from vehicle- or CM-treated experimental pups with intestinal injury and from healthy dam-fed controls and lysed in RIPA buffer. Fifty µgs of lysates were used to determine intestinal TNF-α levels using the xMAP technology. Data (n≥5) are presented as mean ± SE. “*” depicts p<0.05 compared to the vehicle group by one-way ANOVA with Bonferroni correction.

To investigate the effect of CM on inflammatory cytokine production, the pro-inflammatory cytokine TNF-α, an NF-κB target elevated in clinical and animal experimental NEC, in intestinal homogenates was next measured. As shown in Fig. 4, healthy dam-fed control pups had the lowest intestinal TNF-α levels. Increased TNF-α was detected in vehicle-treated diseased experimental model pups. Lp, La/Bi, and La/Bi/Lp CM significantly decreased TNF-α levels in pups exposed to the experimental intestinal injury model.

### Combined conditioned media from Lp, La, and Bi protected intestinal barrier function and maintained the tight junction (TJ) protein ZO-1 at TJs

**Figure 5:**
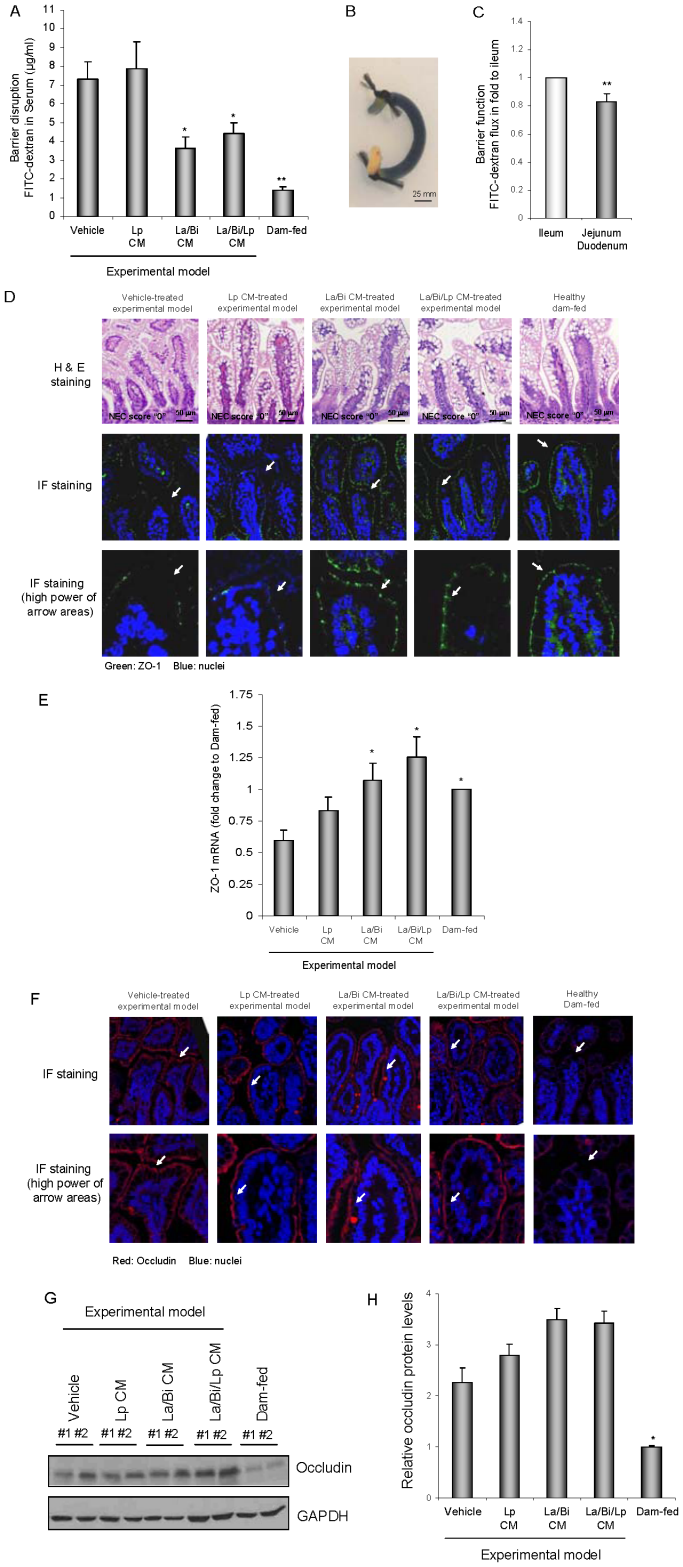
CM from *L. plantarum*, *L. acidophilus*, and *B. infantis* protected intestinal barrier integrity. ***A.*** Combined La/Bi and La/Bi/Lp CM protected intestinal barrier function. Experimental pups were fed with formula plus vehicle or CM from La, Bi, and Lp and stressed to induce intestinal injury. *In vivo* barrier function was determined in all surviving experimental as well as healthy dam-fed pups at the end of the animal experiment on day 5. Data from several animal experiments (n≥14) are presented as mean ± SE. Higher levels of the tracer FITC-dextran in blood indicate poorer intestinal barrier function. ***B.*** An image of sutured intestinal explant loop for *ex vivo* loop/barrier function assay. The loop was filled with trypan blue for demonstration. ***C.*** Ileum exhibited poor barrier function compared to jejunum/duodenum in three-day old experimental pups. Explants of ileum and jejunum/duodenum were harvested from euthanized rat pups, flushed, filled with dialyzed 10 kDa FITC-dextran (1 mg/mL in PBS), and ligated to form closed loops. Explanted loops were placed in PBS and fluorescence in PBS was measured at 30, 60, and 90 minutes. Barrier integrity of paired ileum and jejunum/duodenum from experimental pups (n = 5) at all time points (n = 15) was measured and compared to ileum. ***D.*** Combined La/Bi and La/Bi/Lp CM protected the localization and expression of the TJ protein ZO-1 in the intestine in experimental intestinal injury model. Intestines from animals used in ***A.*** were H&E and IF stained for ZO-1 and nuclei and then subjected to confocal microscopy. Representative immunofluorescence staining shows preserved expression and localization of ZO-1 at the TJs of healthy, La/Bi and Lp/La/Bi CM-treated experimental intestines but not in the vehicle- or Lp CM-treated ones. H&E staining demonstrates intestinal injury score “0” (disease free) for all sections. ***E.*** Combined La/Bi and La/Bi/Lp CM protected intestinal ZO-1 mRNA expression in experimental pups. Total RNA was isolated from intestine and reversed transcribed to obtain cDNA, which was then subjected to TaqMan real-time PCR to determine expression of ZO-1 and GAPDH mRNA in triplicate (n = 3). Normalized ZO-1 mRNA levels are presented in fold to dam-fed controls. ***F.*** CM from La, Bi, or La did not protect expression of the TJ protein occludin in the intestine in experimental intestinal injury. Intestines used as in ***D.*** were IF stained for occludin and nuclei and then subjected to confocal microscopy. Representative immunofluorescence staining shows increased occludin expression in the intestinal injury model compared to the healthy dam-fed control and all CM failed to normalize occludin levels to that in healthy controls. ***G.*** CM did not normalize occludin expression in experimental pups to that of healthy controls. Intestines from vehicle- and CM-treated experimental pups surviving to the end of the experiments (disease free) and healthy dam-fed pups were lysed and subjected to immunoblotting for occludin and GAPDH. Protein levels were quantified by densitometry using ImageJ software and GAPDH normalized occludin levels are presented as mean ± SE in ***H.*** “*” and “**” depict p<0.05 and 0.001, respectively, by t-test in ***C*** and compared to the vehicle group by one-way ANOVA with Bonferroni correction in ***A***, ***E***, and ***H***.

NEC is associated with intestinal barrier disruption [8], [37], [57]. To determine if CM had a cytoprotective effect in addition to an anti-inflammatory effect, intestinal barrier function was assessed by transmucosal transport of fluorescent FITC-dextran (10 kDa) to blood in surviving pups at the end of the animal experiments [49]. Surviving pups were used as these pups all have an intestinal injury histologic score “0”, thus alterations in barrier function seen are not confounded by alterations induced by tissue necrosis associated with disease state. The healthy dam-fed control pups had the lowest level of FITC-dextran in the blood (intact barrier function) whereas the vehicle-treated experimental model pups had the highest levels of FITC-dextran in the blood (poorer barrier function) (Fig. 5A). La/Bi and La/Bi/Lp CM significantly decreased blood FITC-dextran levels, indicating protection of barrier function by these CM in the experimental intestinal injury model. In contrast to combined CM, Lp CM failed to protect barrier function.

The ileum of the intestine is the classically described affected area in NEC. An *ex vivo* loop assay was conducted to measure and compare the barrier integrity of paired ileum and non-ileal regions in the intestine, jejunum and duodenum, from experimental rat pups. Our result shows that ileum is leakier than jejunum/duodenum in the pups (Fig. 5B and C).

Intestinal barrier function can be regulated by both TJ structure and proper TJ protein expression. Others and we have previously shown that occludin is up-regulated while expression and localization of ZO-1 are lost during the development of NEC [20], [49], [58]. Since La/Bi and La/Bi/Lp CM protected intestinal barrier function in the experimental model (Fig. 5A), immunofluorescence staining for ZO-1 and occludin in the ileum was evaluated in intestines from the same animals used for the *in vivo* barrier function assay. Immunofluorescence staining revealed normal TJ structure and ZO-1 protein expression predominantly at the TJs in healthy dam-fed controls. In contrast, ZO-1 staining at TJs was lost in ileal villi of vehicle-treated experimental pups (Fig. 5D). This alteration in ZO-1 by experimental stress was prevented by feeding pups formula supplemented with La/Bi and La/Bi/Lp CM but not by Lp CM. Quantitative real-time RT-PCR shows protected ZO-1 mRNA expression in La/Bi and La/Bi/Lp CM-treated groups (Fig. 5E), supporting the staining results that show combined CM protection of ZO-1 expression in the experimental model. H&E staining confirmed no histological damage in all the pups used for this experiment (Fig. 5D, top panels), suggesting that the loss of ZO-1 at the TJ was not due to intestinal necrosis. Immunoflourescence staining and immunoblotting for occludin show increased occludin in the intestine of vehicle-treated pups exposed to the experimental model compared to healthy controls (Fig. 5F, G, and H). All CM failed to normalize occludin levels to that in healthy controls, demonstrating that combined CM protection of barrier function is not mediated through regulation of occludin.

## Discussion

Using an experimental NEC-like intestinal injury model, we explored the protective effects of different combinations of conditioned media (CM) from *L. plantarum* (Lp), *L. acidophilus* (La), and *B. infantis* (Bi) on pathological mechanisms associated with NEC. We have utilized the most widely used rodent model of NEC in which pups were subjected to risk factors for human NEC (prematurity, formula feeding, and hypoxia-ischemia) and many of the clinical and pathological changes in the immature rat pups were similar to those found in humans (the abdomen was distended; blood was detected in the stool, and the ileum and proximal colon were the most affected parts of the intestine [37]. We recognize that animal experiments can only be “models” of human diseases, thus the pathophysiology of a rodent model may be different from what is seen in humans. However, we used this model to understand and test effects of CM on pathophysiologic mechanisms associated with intestinal injury in NEC.

The efficacy of combined administration of live La and Bi in preventing NEC has been demonstrated in prospective clinical studies. A study led by Hoyos demonstrated that administration of these two live bacteria decreased the incidence of NEC from 6.6% to 2.9% in all newborns admitted to a neonatal intensive care unit in Colombia [26]. Another subsequent study by Lin et al again demonstrated that these organisms significantly reduced NEC incidence in preterm infants <1500 grams [27], [59]. These studies, while documenting possible beneficial clinical effect did not investigate mechanisms for the protective effect of the probiotics used and did not describe a rationale for the specific probiotic organisms selected. Little is known about how these organisms affect the intestine. As these organisms have already been shown to have clinical benefit in combination, we tested the effect of CM from these organisms in combination rather than individually and studied the underlying mechanisms. Previous studies of CM from these organisms had suggested cytoprotective effects [32], [35]. As NEC has specifically been associated with inflammation via NF-κB signaling, we additionally tested the effect of Lp CM which we have previously shown influences NF-κB activation through proteasome inhibition *in vitro* and *ex vivo*. Lp, La/Bi, and La/Bi/Lp CM all decreased intestinal injury incidence in our animal model; however only combined CM significantly protected immature pups from intestinal injury and triple combined CM elicited the most protective effect.

The transcription factor nuclear factor kappaB (NF-κB), a key inflammatory mediator in NEC, activates transcription of a wide variety of cytokines central to the inflammatory response. In its resting state, NF-κB dimers are restricted to the cytoplasm by binding to the inhibitory kappa B proteins (IκB). Extracellular stimuli trigger activation of IκB kinase (IKK), which in turn phosphorylates IκBα, targeting it for degradation by the ubiquitin-proteasome pathway. The NF-κB thus liberated translocates to the nucleus, resulting in transcription of a broad spectrum of pro-inflammatory cytokines. Specific inhibition of the proteolytic activity of the proteasome prevents NF-κB activation and results in the accumulation of ubiquitinated forms of IκBα, indicating an important role of the protesome in regulating IκBα protein expression [60]. We have previously published that Lp CM inhibits NF-κB via proteasome inhibition *in vitro* and *ex vivo* [32] and thus focus on combined condition media modulation of IκBα through this mechanism. We observed higher chymotrypsin-like activity of the proteasome in liver in untreated compared to Lp and La/Bi CM-treated animals, indicating that Lp CM and La/Bi CM inhibited proteasome activity *in vivo* when administered to rats by oral gavage. The proteasome activity was measured in liver rather than in intestine in our animal model due to poor feasibility of isolating proteasomes from the small intestinal protein fractions from the 4-gm rat pups used in the model. As colitis globally affects proteasome activity in gastrointestinal as well as non-gastrointestinal tissues [56], higher chymotrypsin-like activity of proteasome is likely to be observed in intestine in untreated experimental animals, which is supported by and correlated with the decreased IκBα levels and higher TNF-α levels in the intestine (Fig. 2 and 4). However our data does not definitively demonstrate the effect of CM on intestinal proteasomes.

Other studies of probiotics in the pathophysiology of NEC have also demonstrated the potential importance of NF-κB pathway suppression in the mechanism of protection. Among other probiotics tested, *Lactobacillus reuteri* has been shown to reduce NEC incidence [61]. This probiotic also suppressed NF-κB signaling but through a mechanism different from proteasome inhibition. Orogastric administration of live *L. reuteri* decreased IκBα phosphorylation as well as NF-κB and Toll-like receptor-4 (TLR-4) protein expression. It has been shown that changes in intestinal TLR preceded histological injury in a rat model of NEC [62]. Thus, *L. reuteri* attenuates inflammation by directly and indirectly suppressing inflammatory signaling mediators to protect against NEC. In a separate study, administration of *Saccharomyces boulardii lyo*, also inhibited inflammatory pathways by suppressing expression of TLRs as well as by preserving caveolin-1 and NOS in the developing gut [63], [64]. Whether the relevant bioactive factor responsible for the effects seen in these studies is a component of the bacteria itself or a factor released by the bacteria remains unknown. It is likely that combinations of CM from organisms other than those we tested will also have anti-inflammatory and barrier protective effects, and thus may be similarly effective. The anti-inflammatory effect of the bacterium-free components in the CM we used appears to be mediated through protected IκBα expression and suppressed NF-κB signaling possibly associated with decreased proteasome activity. It is a next natural step to characterize the bioactive components in the CM responsible for the beneficial effects. Metagenomic studies of the microbiota suggest that functions of organisms are of greater importance than taxonomy. For the preterm infants, we expect that understanding the critical effects of microbiota necessary for enhanced development of the immature host that then may be induced by various interventions to protect against NEC, will be of greater importance than identification of individual optimal probiotic strains.

In addition to inducing an inflammatory response, proinflammatory cytokines regulated by NF-κB activation are known to induce barrier disruption. TNF-α has been shown to be elevated in NEC [65] and to increase intestinal epithelial permeability both *in vitro* and *in vivo* [66]–[68]. Thus, CM inhibition of NF-κB and pro-inflammatory cytokine production may also contribute to preservation of barrier function. However, in addition to inflammation-mediated barrier disruption, barrier function is regulated by levels and function of TJ proteins as well as by cellular responses such as apoptosis and autophagy. One of the factors that maintains barrier integrity is the tight junction, a connection formed by/between intestinal epithelial cells at their most apical area. Disruption of the TJ can lead to hyper-permeability of the gut with increased bacterial translocation and a resulting inflammatory response predisposing the host to NEC. Tight junction trans-membrane proteins, including occludin, claudins, and junctional adhesion molecules, as well as cytoplasmic proteins such as zonula occludens (ZO-1, ZO-2, and ZO-3) [69], work in concert to form physical connections between epithelial cells and confer basic barrier properties. ZO-1 contains protein-binding domains for interaction with other tight junction-associated proteins and the peri-junctional actin ring [70], [71] and serves as a functionally critical linker between the TJ and the actin cytoskeleton to maintain TJ integrity. Our data demonstrate that combined La/Bi and La/Bi/Lp CM preserved ZO-1 protein expression at the tight junction as well as mRNA expression, correlating with preservation of barrier function by these combined CM, whereas Lp CM had no effect. Occludin is another TJ protein that has been shown to be up-regulated in experimental NEC [20], [58]. Consistently, increased occludin was detected in experimental NEC by immunofluorescence staining and immunoblotting (Fig. 5F, G, and H). All CM failed to normalize occludin levels to that in healthy controls, indicating combined CM protection of barrier function was not mediated through regulation of occludin. We have previously shown that preservation of the TJ protein ZO-1 at TJs is important for barrier protection in experimental NEC [49] and these observations may explain why Lp CM, with its lack of effect on TJ proteins, failed to protect barrier function. Our data indicate that both La/Bi and La/Bi/Lp CM maintained the TJ structure and ZO-1 protein expression at TJs and protected barrier function.

Ileum of the intestine is the classically affected area in NEC and injury in ileum has been used to evaluate the severity of intestinal injury in the model used [72], [73]. It is possible that CM changed barrier integrity across both ileal and non-ileal regions of the intestine such as jejunum and duodenum. We have now performed an *ex vivo* loop assay to measure and compare the barrier integrity of paired ileum and jejunum/duodenum from experimental rat pups (Figure 5B and C). Our results show that ileum is leakier than jejunum/duodenum (5 animals, three time points of FITC-dextran flux; n = 15, Figure 5C). As the ileum is the leakier area compared to jejunum and duodenum and CM protected tight junction structure and ZO-1 localization at tight junctions in ileum, CM is unlikely to act only in non-ileal regions to protect barrier function. Although the *in vivo* barrier function assay does not measure gut permeability specifically in ileum, it allows us to collect data with more biological relevance and in sum, CM protected barrier function *in vivo*.

In diseased pups with severely damaged intestine, all three different CM preserved IκBα levels by IHC and immunoblotting. However, CM with only anti-inflammatory effects was insufficient to significantly protect against intestinal injury, as Lp CM decreased intestinal injury incidence only to a minor degree. Administration of combined La/Bi and La/Bi/Lp CM further reduced NEC-like intestinal injury incidence, suggesting an added benefit from a combination of anti-inflammatory and cytoprotective characteristics. These results suggest a synergy between CM from different organisms and the potential importance of use of organisms with specific desired effects for therapeutic or preventative purposes.

Recent reports have demonstrated that CM or soluble proteins in CM from *Lactobacillus rhamnosus* GG (LGG), one of the best studied *Lactobacillus* strains used in clinical trials for inflammatory bowel diseases, have cytoprotective effects and can promote intestinal epithelial homeostasis [33], [74]–[76]. From the same studies, it appears that the active soluble factors released by the LGG are proteins or peptides. From our studies, preliminary characterization of the NF-κB signaling-inhibiting bioactive factors in Lp CM indicates that they are of small molecular weight (less than 10 kDa), heat stable, and pepsin protease resistant, indicating they are likely small molecules such as bacterial metabolites, and not proteins [32]. Identification and purification of CM-derived soluble factors that regulate intestinal epithelial cell inflammation and barrier function would provide a molecular basis for therapeutic application of probiotic bacterial products for inflammation-mediated intestinal disorders. Thus, isolating the relevant factors in La/Bi CM used in this study is a natural next step however beyond the scope of the current work.

It has been shown that the mechanism of protection conferred by probiotics depends upon the specific probiotic strain and disease model used in investigation [20], [59], [77]. In the current study, Lp, La/Bi, and La/Bi/Lp CM elicited different magnitudes of anti-inflammatory and cytoprotective effects. As NEC is a multi-factorial disease, agents such as Lp CM that are able to preserve IκBα and suppress inflammatory signaling but do not protect barrier function may not be as protective as La/Bi and La/Bi/Lp CM that preserve IκBα as well as barrier integrity in the intestinal injury model. The La/Bi/Lp CM combination exhibited the most protective effect, suggesting that a multi-strain CM approach combining different mechanisms of action that complement each other provides more effective protection against disease. A multi-strain strategy which tries to more closely recapitulate an intestinal microbial ecosystem has been proposed as a potentially more effective treatment for several other gastrointestinal diseases [10] and CM derived from multiple strains may similarly prove to provide more benefit.

NEC is thought to be a disease of the interaction between the premature gut and the intestinal microbiota, thus it has been suggested that means of optimizing bacteria colonization may decrease disease incidence. However, concerns persist regarding the safety of administration of large numbers of live organisms to the relatively immunocompromised preterm infants. There is no clear quality control of probiotics from industry and the probiotic industry has recently been criticized in Europe for lack of substantiation of multiple health claims [78]. A study by Drago L. et al showed only 4 of 13 probiotic products (31%) in the USA market in 2009 were in accordance with label claims in terms of quantity of viable bacteria, identification of species, and cross contamination by species not on the label [79]. The report suggests the need for adequate control of probiotic production as well as periodic screenings by competent organizations to monitor the effect of storage on product quality. Other concerns regarding the use of probiotic therapy include the difficulty determining the viability of bacteria in the gastrointestinal tract, and the risk of infectious complications described in association with probiotic use in very young and sick patients [31], [80]. Therefore, one approach to address these questions may be to use probiotic bacterial-derived factors as novel therapeutic agents for treatment of NEC and perhaps other inflammation-related disorders such as inflammatory bowel diseases. The use of bacteria-free bioactive products synthesized by probiotic bacteria, rather than the live bacteria themselves, may be a strategy to improve safety of probiotic-based treatment by eliminating risk of infection associated with the use of live organisms.

In summary, this study using an animal model with intestinal injury similar to NEC provides insight into the mechanisms behind the protective effects of select organisms and demonstrates efficacy of use of combined CM from these organisms in ameliorating the mechanisms associated with the pathophysiology of NEC. In addition, this current report also provides evidence for isolation and use of the beneficial bioactive factors in the CM from the organisms. Using bioactive factors from probiotic CM could ultimately culminate in the development of novel therapeutic agents as purified factors which can be administrated in a safer and reproducible, pharmacologic manner to treat or prevent NEC in at risk infants.
